# Life-threatening ventricular arrhythmia due to silent coronary artery spasm: usefulness of I-123 metaiodobenzylguanidine scintigraphy for detecting coronary artery spasm in the era of automated external defibrillators: a case report

**DOI:** 10.1186/1752-1947-9-26

**Published:** 2015-02-06

**Authors:** Bunji Kaku, Shoji Katsuda, Tomio Taguchi

**Affiliations:** Division of Cardiovascular Medicine, Toyama Red Cross Hospital, 2-1-58 Ushijima-honmachi, Toyama, 930-0859 Japan

**Keywords:** AED, MIBG scintigraphy, Silent coronary artery spasm, Ventricular fibrillation

## Abstract

**Introduction:**

Cardiac arrhythmia is sometimes life-threatening, and automated external defibrillators are presently used in some countries. Coronary artery spasm is one of the primary causes of life-threatening arrhythmia. In general, chest symptoms are key indicators of possible coronary artery spasm; however, if chest symptoms are not present, clinicians may not suspect this disease. We encountered a patient who had recovered from ventricular fibrillation treated by using an automated external defibrillator, and silent coronary artery spasm was considered to be the cause of this life-threatening arrhythmia. In this case, I-123 metaiodobenzylguanidine scintigraphy was a useful screening tool for a silent coronary artery spasm.

**Case presentation:**

A 72-year-old Japanese man was transferred to our hospital after recovering from ventricular fibrillation treated by using an automated external defibrillator. He had never complained of chest symptoms previously. Decreased uptake of I-123 metaiodobenzylguanidine was observed in the inferolateral and anteroseptal walls of the left ventricle. A spasm provocation test of the coronary artery was performed, and silent coronary artery spasm was diagnosed as the underlying disease.

**Conclusion:**

Non-invasive I-123 metaiodobenzylguanidine scintigraphy was a useful screening tool for silent coronary artery spasm as a possible cause of cardiopulmonary arrest in a patient with no chest symptoms.

## Introduction

Arrhythmias can be life-threatening, and their treatment with an automated external defibrillator (AED) has become prevalent in some countries [1]. It is important to identify the underlying disease [2, 3]. Coronary artery spasm is a major cause of life-threatening arrhythmia. In general, myocardial ischemia due to coronary artery spasm is associated with chest pain or discomfort that occurs during rest or ordinary exercise, and this symptom is a key indicator of possible coronary artery spasm. However, if there are no chest symptoms, clinicians may not suspect this disease. We encountered a patient who had recovered from ventricular fibrillation treated by using an AED, and silent coronary artery spasm was considered to be the cause of this life-threatening arrhythmia. In this case, I-123 metaiodobenzylguanidine (MIBG) scintigraphy was a useful screening tool for a silent coronary artery spasm.

## Case presentation

**Figure 1:**
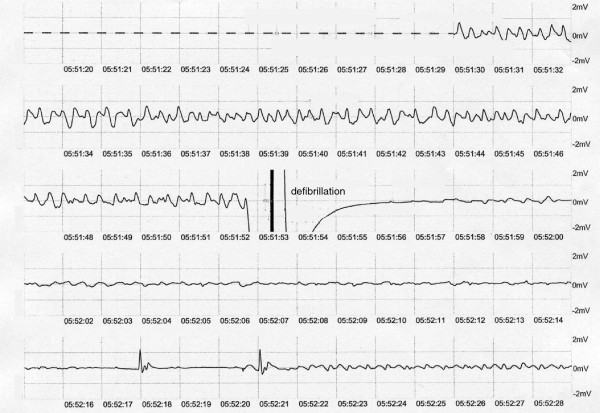
**Electrocardiogram obtained during the use of an automated external defibrillator.** Ventricular fibrillation was detected, and defibrillation therapy was performed using an automated external defibrillator.

**Figure 2:**
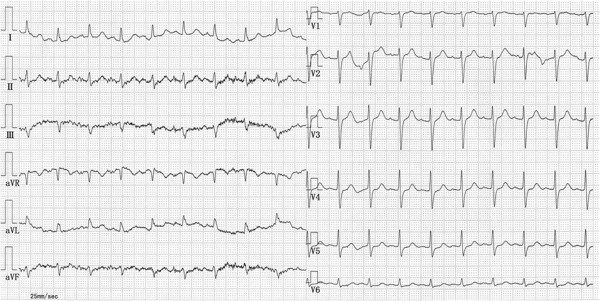
**Electrocardiogram obtained soon after admission.** No remarkable ST-T abnormality was found.

**Figure 3:**
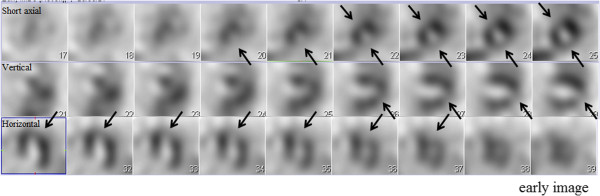
**I-123 metaiodobenzylguanidine scintigram recorded 20 days after the cardiac event.** Decreased uptake of I-123 metaiodobenzylguanidine was observed in the inferolateral and anteroseptal walls of the left ventricle (arrows).

**Figure 4:**
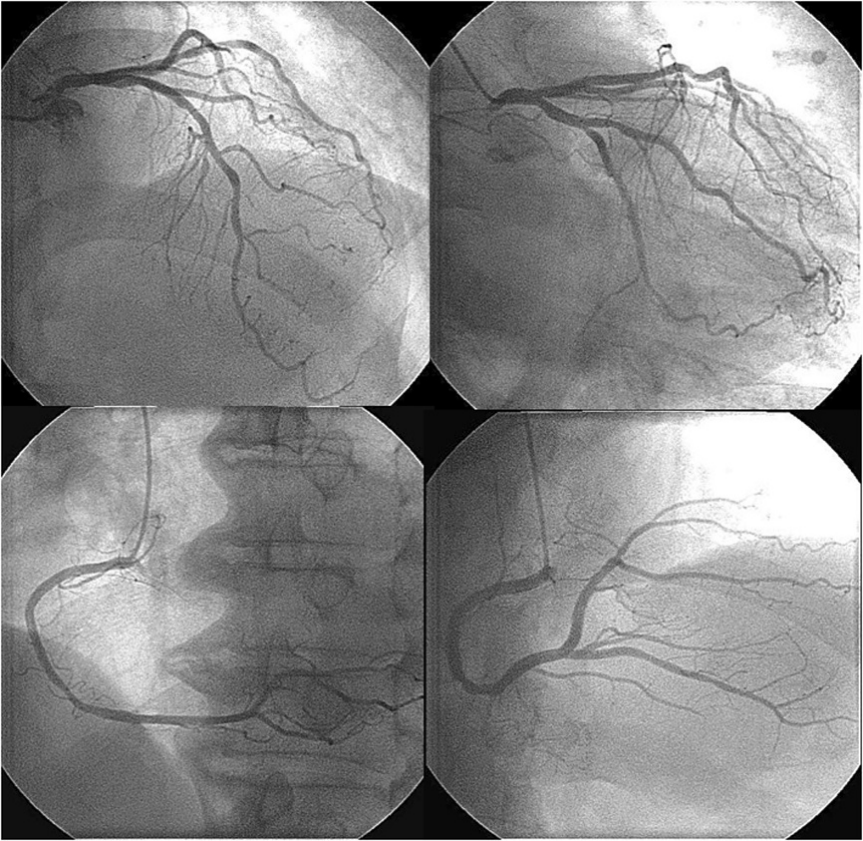
**Coronary angiogram does not show any severe organic stenosis in either the right or left coronary artery.**

**Figure 5:**
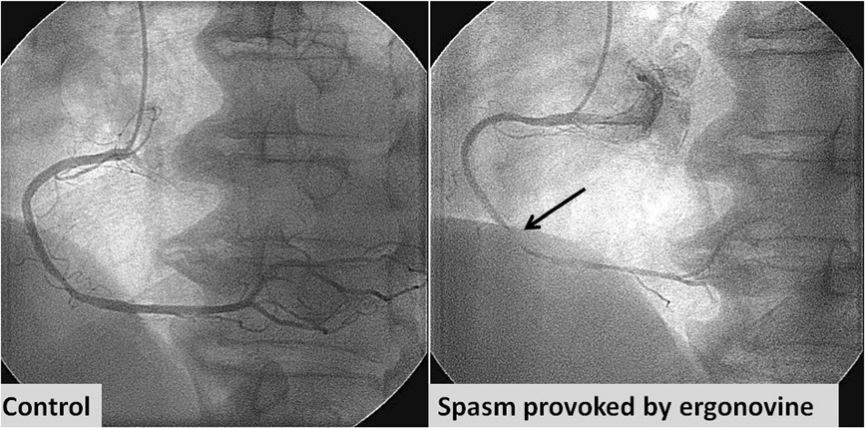
**Coronary angiogram obtained during the spasm provocation test.** After intra-coronary infusion of low-dose ergonovine (10μg), a severe coronary artery spasm was provoked in the right coronary artery without any chest symptoms (arrow).

**Figure 6:**
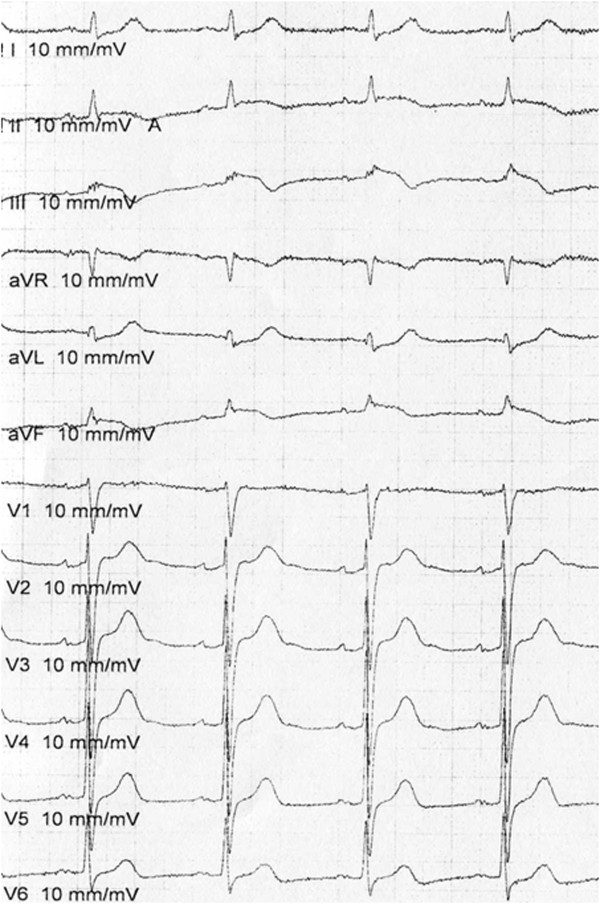
**Electrocardiogram obtained during the spasm provocation test.** ST elevation in the inferior leads was observed.

A 72-year-old Japanese man was transferred to our hospital after recovering from ventricular fibrillation treated by using AED. He had suddenly collapsed during a walk without any symptoms in the early morning, and an AED had been used to perform defibrillation after a bystander had performed cardiopulmonary resuscitation (Figure 1). Although the patient was in a coma at admission, his consciousness markedly improved after he received hypothermal therapy and brain-protecting drugs. He had been medicated for hypertension in another hospital and had never complained of chest symptoms. Several months before this event, he had experienced transient syncope without chest symptoms. At that time, no abnormal findings were detected in a treadmill exercise test or during Holter recording, which had been performed at another hospital. After he was admitted to our hospital, electrocardiography showed no abnormality (Figure 2). Further, echocardiography and cardiac magnetic resonance imaging (MRI) showed no wall motion abnormality and no structural heart disease. Delayed enhancement in the myocardium was also not observed in the cardiac MRI. Tc-99m myocardial single-photon emission computed tomography (SPECT) showed no perfusion abnormality, and quantitative gated SPECT revealed a left ventricular ejection fraction value of 66%. Holter recording and continuous electrocardiographic monitoring performed for >2 weeks showed no apparent ventricular arrhythmia. Myocardial imaging using I-123 MIBG was performed 20 days after admission. With the patient in the supine position at rest, 111MBq of I-123 MIBG was intravenously administered. An initial image was obtained 20 minutes after the intravenous injection, and a delayed scan was obtained 4 hours later. Decreased uptake of I-123 MIBG was observed in the inferolateral and anteroseptal walls of the left ventricle (Figure 3); therefore, cardiac catheterization was planned. Coronary angiography did not reveal any severe organic stenosis in either the right or left coronary artery (Figure 4). A spasm provocation test was subsequently performed. Ergonovine (10μg) was initially injected into the right coronary artery for 4 minutes. After the injection, severe coronary artery spasm was induced with ST elevation in the inferior leads (Figures 5 and 6). Despite the ST elevation, the patient did not complain of any chest symptoms. Moreover, to try to prevent secondary brain damage due to malignant ventricular arrhythmia during the ergonovine provocation test, an intracoronary injection of nitrate (3mg of isosorbide dinitrate) was provided to resolve the provoked coronary artery spasm after the ST elevation was confirmed. Silent coronary artery spasm was diagnosed. Administration of a calcium channel blocker and nitrate was initiated, and a cardioverter defibrillator was implanted to provide secondary prevention.

## Discussion

In the era of AEDs, the rescue rate from cardiopulmonary arrest due to life-threatening arrhythmia has improved [1]. It is important to identify the underlying cause of arrhythmia. Non-invasive methods, such as echocardiography or cardiac MRI, are useful tools for detecting underlying structural heart disease. Even organic coronary arterial stenosis can be detected by non-invasive computed tomography (CT) angiography. In contrast, even in the absence of structural heart disease, transient myocardial ischemia may be the triggering mechanism for fatal ventricular arrhythmia in some patients with cardiac arrest [2, 3]. Spasm of the epicardial coronary arteries is one of the important mechanisms of transient myocardial ischemia and can cause life-threatening arrhythmia [2, 3]. Kobayashi and colleagues reported that coronary artery spasm was diagnosed in approximately 7% of patients resuscitated after out-of-hospital cardiac arrest (OHCA) caused by cardiovascular disease [4]. Takagi and colleagues similarly documented coronary artery spasm in 22 (6.0%) of 365 patients resuscitated from OHCA of cardiac origin [5]. In general, chest symptoms due to coronary artery spasm are characterized by resting anginal attacks occurring mainly at night and in the early morning. However, in some patients, coronary artery spasms without any chest symptoms have been reported, and this silent myocardial ischemia due to coronary artery spasm may cause life-threatening ventricular arrhythmia and heart failure [2, 3, 6, 7]. Myerbrug and colleagues found silent ischemic events due to coronary artery spasm that were associated with life-threatening ventricular arrhythmia in 5 (1.4%) of 356 survivors of OHCA [6]. This spasm-related ventricular arrhythmia is difficult to induce by programmed ventricular stimulation during electrophysiological testing [6, 8]. Because of its rarity and asymptomatic features, coronary artery spasm may sometimes be difficult to diagnose in this situation. Furthermore, in many cases, invasive cardiac catheterization, including the spasm provocation test, is still necessary for an accurate diagnosis of coronary artery spasm. If patients do not complain of any chest symptoms, it is possible that clinicians will not consider an invasive ergonovine provocation test.

In patients with coronary artery spasm, myocardial imaging using I-123 MIBG is generally performed for two purposes: as a diagnostic tool [9–12] and for the prediction of prognosis [13]. Some non-invasive methods have been used to detect coronary artery spasm; in these methods, I-123-β-methyl-iodophenyl-pentadecanoic acid (BMIPP) or MIBG have been proposed as useful tracers for the detection of myocardial damage due to coronary artery spasm [9–11]. Although the specificity of MIBG imaging is lower than that of BMIPP imaging, the sensitivity is much higher for the identification of coronary artery spasm [9]. Watanabe and colleagues reported that the sensitivity was 96% and specificity was 55% for the determination of coronary artery spasm by MIBG SPECT [9]. Taki and colleagues similarly reported that the positive and negative predictive values of MIBG SPECT for coronary artery spasm were 83% and 81%, respectively [11]. Furthermore, Sakata and colleagues reported the superiority of regional washout rate analysis of MIBG imaging using a bull’s-eye map than visual analysis or quantitative analysis for detecting patients with vasospastic angina with sporadic attacks [12]. The same investigators also stated that using the heart-to-mediastinum ratio and washout rate determined by MIBG imaging had prognostic value in a patient with vasospastic angina [13].

In our patient, although the precise mechanism of decreased uptake of I-123 MIBG in the inferolateral and anteroseptal walls of the left ventricle was not clear, it is natural to assume that severe myocardial ischemia caused by coronary artery spasm resulted in transient myocardial damage and sympathetic denervation. Ventricular arrhythmias were not detected during the spasm provocation test, possibly because nitrate was administered after provocation of the coronary artery spasm. By the use of hypothermal therapy and brain-protecting drugs, our patient’s brain function unexpectedly improved. It was also important to avoid secondary brain damage due to malignant ventricular arrhythmia during the ergonovine provocation test. To try to prevent the development of this serious condition, we administered nitrate without delay.

Cardiac sympathetic denervation is associated with ventricular arrhythmia [14], and denervated but viable myocardium may be hypersensitive to catecholamine [15]. Although it is still controversial, the extent of I-123 MIBG–perfusion mismatch is thought to be related to the occurrence of ventricular arrhythmias in some settings of cardiac diseases [16, 17]. In patients with acute coronary syndrome, I-123 MIBG–perfusion mismatch size that indicates the extent of denervated but viable myocardium correlates with a prolongation of ventricular repolarization and late potentials in the depolarization phase [16]. In patients with heart failure, I-123 MIBG–perfusion mismatch size is significantly associated with appropriate implantable cardioverter defibrillator therapy [17]. Although malignant ventricular arrhythmia was not reproduced in the spasm provocation test in the present case, I-123 MIBG–perfusion (Tc-99m myocardial SPECT) mismatch was apparent, and this fact supported the suspicion that silent coronary artery spasm might be the cause of ventricular fibrillation.

In general, abnormal MIBG uptake is associated with abnormal left ventricular wall motion. However, abnormal MIBG uptake is observed in cases in which left ventricular wall motion is not impaired [9]. In our patient, abnormal left ventricular wall motion was not detected at admission, and abnormal MIBG uptake was observed even 20 days after the cardiac event. On the basis of these abnormal MIBG imaging findings, we performed an invasive spasm provocation test, and silent coronary artery spasm was accurately diagnosed.

## Conclusion

Non-invasive MIBG imaging was a useful screening tool for a life-threatening silent coronary artery spasm in a patient without chest symptoms.

## Consent

Written informed consent was obtained from the patient for the publication of this case report and any accompanying images. A copy of the written consent is available for review by the Editor-in-Chief of this journal.

## Abbreviations

AED: Automated external defibrillator
BMIPP: I-123-β-methyl-iodophenyl-pentadecanoic acid
CT: Computed tomography
MIBG: I-123 metaiodobenzylguanidine
MRI: Magnetic resonance imaging
OHCA: Out-of-hospital cardiac arrest
SPECT: Single-photon emission computed tomography.
